# Chromium Monitoring in Water by Colorimetry Using Optimised 1,5-Diphenylcarbazide Method

**DOI:** 10.3390/ijerph16101803

**Published:** 2019-05-21

**Authors:** Annija Lace, David Ryan, Mark Bowkett, John Cleary

**Affiliations:** 1 EnviroCORE, Department of Science and Health, Institute of Technology Carlow, Kilkenny Road, Co. Carlow R93 V960, Ireland; david.ryan@itcarlow.ie (D.R.); john.cleary@itcarlow.ie (J.C.); 2 TE Laboratories Ltd. (TelLab), Loughmartin Business Park, Tullow, Co. Carlow R93 N529, Ireland; mbowkett@tellab.ie

**Keywords:** chromium, colorimetric methods, environmental monitoring, 1,5-diphenylcarbazide, microfluidics

## Abstract

Chromium contamination of drinking water has become a global problem due to its extensive use in industry. The most commonly used methods for chromium detection in water are laboratory-based methods, such as atomic absorption spectroscopy and mass spectroscopy. Although these methods are highly selective and sensitive, they require expensive maintenance and highly trained staff. Therefore, there is a growing demand for cost effective and portable detection methods that would meet the demand for mass monitoring. Microfluidic detection systems based on optical detection have great potential for onsite monitoring applications. Furthermore, their small size enables rapid sample throughput and minimises both reagent consumption and waste generation. In contrast to standard laboratory methods, there is also no requirement for sample transport and storage. The aim of this study is to optimise a colorimetric method based on 1,5-diphenylcarbazide dye for incorporation into a microfluidic detection system. Rapid colour development was observed after the addition of the dye and samples were measured at 543 nm. Beer’s law was obeyed in the range between 0.03–3 mg·L^−1^. The detection limit and quantitation limit were found to be 0.023 and 0.076 mg·L^−1^, respectively.

## 1. Introduction

Environmental contamination of chromium has become a global concern because of its major role in industry [1,2,3]. Chromium is widely used in leather tanning, electroplating, paint manufacture, wood treatment, metallurgy, and mining [4,5,6,7]. Significant amounts of chromium are introduced into the environment through poorly regulated disposal of chromium containing waste [8]. As a result, chromium concentrations in surface and drinking water can exceed the World Health Organization’s maximum allowable concentration of 0.05 mg·L^−1^ [9,10].

In the environment, chromium can be most commonly found in two oxidation states—hexavalent (Cr VI) and trivalent (Cr III) [11]. Redox and pH conditions determine chromium speciation in water. Generally, Cr VI is more abundant under oxidizing conditions, while Cr III dominates under reducing conditions [12,13].

Cr III, at appropriate levels, is beneficial for human health and is involved in lipid and glucose metabolism, whereas Cr VI is toxic [14,15]. Health problems associated with Cr VI exposure include skin rashes, kidney and liver damage, internal haemorrhage, teeth abnormalities, and respiratory ailments, including lung cancer [16,17,18,19].

High Cr VI concentrations have been reported in numerous groundwater sources around the world [20,21,22,23]. One of the most infamous cases of Cr VI pollution took place in Hinkley, California, where concentrations as high as 0.580 mg·L^−1^ were reported in groundwater samples [24]. In Kanpur, India, due to poor waste disposal practices, Cr VI concentration reached 16.3 mg·L^−1^ [25]. In Leon Valley, Mexico, Cr VI concentrations were found to have reached 50 mg·L^−1^ due to industrial runoff [26]. 

Due to the high toxicity of Cr VI and its presence in the environment, effective and reliable monitoring of the species is required. The most commonly used methods for Cr VI detection are atomic absorption spectrophotometry (AAS) and inductively coupled plasma-mass spectrometry (ICP-MS) [27,28]. Although these are powerful and sensitive methods that can detect Cr VI even at trace levels, they are also expensive and require skilled analysts and laborious sampling. Different oxidation states of Cr VI in groundwater can be unstable when exposed to air, temperature fluctuations, and change in pH [29]. Because of this, Cr VI should ideally be detected on site, and therefore, simple, portable, sensitive, and cost-effective methods would be greatly beneficial [30]. 

In recent years, application of microfluidic detection systems for environmental monitoring has gained great interest [31,32,33,34,35]. Microfluidic detection systems are characterised by their small size, which offers the potential for development of portable sensing systems with the ability to analyse samples on a sub-millilitre scale [36]. There are numerous advantages associated with miniaturisation, including reduced sample volume, fast reaction time, and minimised waste production [37]. Additionally, microfluidic detection systems facilitate the development of portable and/or autonomous devices, which can be used on site without the requirement of sample collection and transportation [38]. 

Electrochemical sensors have been used for heavy metal monitoring in water, including Cr VI [39,40,41,42,43]. Methods based on electrochemical detection can achieve very low detection limits and high selectivity [44]. There are, however, numerous limitations associated with electrochemical detection methods that make them difficult to implement for long-term monitoring, such as inability to analyse complex water matrices, sensor drift, high maintenance cost, and biofouling [45]. 

Biofouling is significantly minimised in optical detection systems as they do not require direct contact between the sensor and the sample. Optical detection methods are widely used in microfluidic analysis [46,47,48,49]. Colorimetric methods can be implemented using simple and low-cost detection systems based on light emitting diodes (LEDs) as light source and photodiode detectors, making them suitable for use in portable microfluidic detection systems [50]. 

In recent years, paper-based microfluidic analytical devices (μPADs) have been used for environmental monitoring [51]. μPADs use capillary forces instead of pumps and have low manufacturing costs [52]. Asano et al. used μPADs based on 1,5-diphenyl-carbazide for Cr VI detection in water and obtained an LOD of 30 mg·L^−1^ with a linear range between 40–400 mg·L^−1^ [53]. Idros et al. utilised μPADs for Cr VI detection in water using colorimetric detection with LOD of 0.019 mg·L^−1^ and linear range between 0.019–1.4 mg·L^−1^ [54]. In 2018, Sun et al. developed a Cr VI detection method using a μPADs-based rotational device with linear range between 0.5–10 mg·L^−1^ and LOD of 0.18 mg·L^−1^ [55].

The use of different chromophoric dyes for spectrometric Cr VI detection in water has been described in the literature [56,57,58,59]. Onocke and Sasu developed a method for Cr VI detection in groundwater and industrial waste samples [60]. In this method, Cr VI was reacted with 1,5-diphenylcarbazide (DPC) dye, which, in acidic conditions, forms a purple-coloured species. As a result of a redox reaction, Cr VI is reduced to Cr III, and DPC is oxidised to 1,5-diphenylcarbazone (DPCA). Cr III and DPCA form a purple-coloured species with lambda max of 540 nm [61]. To date, DPC method has not been implemented into an autonomous detection system. 

**Figure 1:**
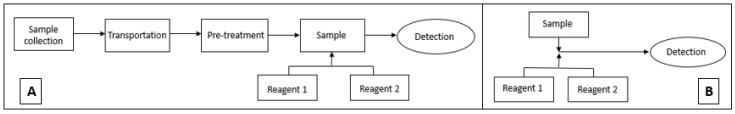
(**A**) Conventional method analysis incorporating multiple steps for Cr VI analysis, (**B**) microfluidic detection-based analysis using modified 1,5-diphenylcarbazide method for Cr VI detection.

The aim of this study is to optimise a DPC method for low-cost and simple incorporation into a microfluidic detection system (Figure 1). The DPC method was chosen because of the intense colour development at 543 nm region. The method’s performance was evaluated in the laboratory on both macro and micro scale. 

## 2. Materials and Methods 

### 2.1. Apparatus

Shimadzu 1800 UV-visible spectrometer was used with Hellma 10 mm and 1 mm quartz cuvettes for the absorbance measurements. Hanna 20 pH meter was used for pH measurements. Varian 820-MS ion-coupled plasma mass spectrometer (ICP-MS) (Varian, Palo Alto, CA, USA) was used to determine Cr VI concentration in the water samples. The settings for the ICP-MS analysis are shown in Table 1. 

### 2.2. Reagents

All chemicals were of analytical grade and purchased from Sigma-Aldrich (Arklow, Ireland), unless otherwise stated. Potassium dichromate (K_2_Cr_2_O_7_), iron chloride 6-hydrate (Fisher Scientific, Leicestershire, UK), magnesium sulphate (MgSO_4_) (Fisher Scientific, Leicestershire, UK), potassium dihydrogen phosphate (KH_2_PO_4_), manganese sulphate 1-hydrate (MnSO_4_·H_2_O), sodium nitrate (NaNO_3_), and chromium chloride hexahydrate (CrCl_3_·6H_2_O) were used to prepare stock solutions at concentration 1000 mg·L^−1^ in double-deionised water. Working standards were prepared by serial dilution. Methanol (CH_3_OH), Tween 20 (C_58_H_114_O_26_), acetonitrile (CH_3_CN) (Lennox, Dublin), hydrochloric acid (HCl), and nitric acid (HNO_3_) (SciChem, Bilston, UK) were used for sample cell cleaning validation. Ascorbic acid (C_6_H_8_O_6_), 1,5-diphenylcarbazide (C_6_H_5_NHNHCONHNHC_6_H_5_), and sodium hydroxide (NaOH) (Sharlab S.L., Barcelona, Spain) were prepared by weighing out an appropriate amount and dissolving it in double deionised water. Sulphuric acid (H_2_SO_4_) (97%) was used to prepare sulphuric acid solutions with various concentrations in double deionised water. Double deionised water was used for dilution of reagents and samples.

### 2.3. Sample Preparation

Cr VI sample (2 mL) was transferred to a glass vial. Sulphuric acid (0.2 M, 1 mL) and 1,5-diphenylcarbazide (0.5% w/v, 1 mL) were added, and the mixture was gently shaken and left for five minutes. The absorbance was measured at 543 nm against reagent blank.

### 2.4. Path Length 

Effect of optical path length on absorbance was investigated in order to simulate the conditions in a microfluidic detection system. The procedure was carried out in standard 10 mm quartz cuvettes and microcuvettes with 1 mm light path for Cr VI solutions with concentrations of 0.1–1.0 mg·L^−1^. The experiment was carried out in triplicate. The average absorbance was calculated, and calibration curves were plotted.

### 2.5. Sample Cell Cleaning Validation

10 mm quartz cuvettes were filled with a solution containing Cr VI and reagents and left to stand for one hour. Double deionised water, 1% hydrochloric acid, 1% nitric acid, methanol, acetonitrile, acetone, Tween 20, and 1% sulphuric acid were used to rinse the cuvettes. The absorbance of cuvettes was measured at 543 nm. The absorbance obtained from different solvents was compared to clean quartz cuvettes (control). All measurements were carried out in triplicate. 

### 2.6. Optimisation of Parameters

#### 2.6.1. pH 

The effect of pH on the method was studied. Sulphuric acid and sodium hydroxide were used to adjust the pH of the double deionised water in which the chromium samples (Cr VI) were prepared. 

#### 2.6.2. Sample/Reagent Ratio

The effect on absorbance of combining the different reagents into a single reagent solution was studied in order to simplify the detection process, which would in turn enable cost effective and uncomplicated microfluidic chip design and fabrication. Sulphuric acid and DPC dye were mixed together in a 1:1 ratio to form a combined reagent, which was then used for the analysis of Cr VI samples. A sample of 2 mL was placed into a glass vial to which 2 mL of combined reagent was added (sample/reagent ratio B). After five minutes, measurements were taken at 543 nm using quartz cuvettes. The absorbance was compared to the original sample/reagent ratio: 1 (sample): 1 (0.4 M sulphuric acid): 1 (DPC), described as sample/reagent ratio A in the results section.

#### 2.6.3. Reagent Stability

The effect of reagent stability on the Cr VI determination was investigated. Firstly, 0.5% DPC dye was used for Cr VI determination over 28 days with fresh 0.4 M sulphuric acid prepared every week. Secondly, 0.5% DPC and 0.4 M sulphuric acid reagent mixture was prepared and used for Cr VI determination over 28 days. Absorbance was measured every week and compared. 

#### 2.6.4. Effect of Different Acid Concentrations

The effect of varying sulphuric acid concentration was studied. Cr VI ranging from 0.1 to 1 mg·L^−1^ were analysed. One-way analysis of variance (ANOVA, single factor) was used to analyse the results obtained. 

### 2.7. Colour Stability

1 mg·L^−1^ Cr VI was analysed using Shimadzu UV-Vis time scan option, the measurements were taken every 60 seconds for 600 min at 543 nm straight after addition of the reagents. The absorbance was plotted against time (min).

### 2.8. Interference

Fe (III), Cr (III), NO_3_, PO_4_, Mg, and Mn were introduced to 1 mg·L^−1^ Cr VI prepared in double deionised water prior to analysis. Tolerance limits of interfering agents were established at those concentrations that do not cause more than 5% error in the absorbance values of Cr VI at 1 mg·L^−1^.

### 2.9. Environmental Samples

Water samples were collected from Killeshin water reservoir, Killeshin, Co. Laois, groundwater well, Co. Laois, and River Barrow at Carlow (Barrow 1) and St. Mullins, Co. Carlow (Barrow 2). All water samples were analysed in triplicate. The sample matrices were analysed using the DPC method in order to determine whether or not Cr VI was present in concentrations detectable by the method. The different water matrices were then spiked with Cr VI (0.1–1 mg·L^−1^) and appropriate dilutions were made. Prior to the analysis, the water samples were filtered, firstly using Whatman grade 1 filter paper and secondly with sterile 0.2 μm syringe filters. The pH of the water samples was adjusted to 2.2. 

### 2.10. Comparison between Optimised DPC Method and ICP-MS 

The optimised DPC method was compared to accredited ICP-MS, which is the gold standard method for heavy metal analysis in water. A calibration curve in a range 0.2–3 mg·L^−1^ was obtained using the optimised DPC method. For control purposes, a 1 mg·L^−1^ standard solution was analysed. The concentration of the sample was calculated from the calibration curve (*y* = 0.2962x − 0.0287). ICP-MS was used to analyse 1 mg·L^−1^ environmental water samples. The concentration for 1 mg·L^−1^ water samples obtained from optimised DPC method and the ICP-MS were compared. Percentage difference was calculated using formula: (Conc. ICP-MS − Conc. DPC method) × 100/Conc. ICP-MS.

## 3. Results

### 3.1. Path Length 

**Figure 2:**
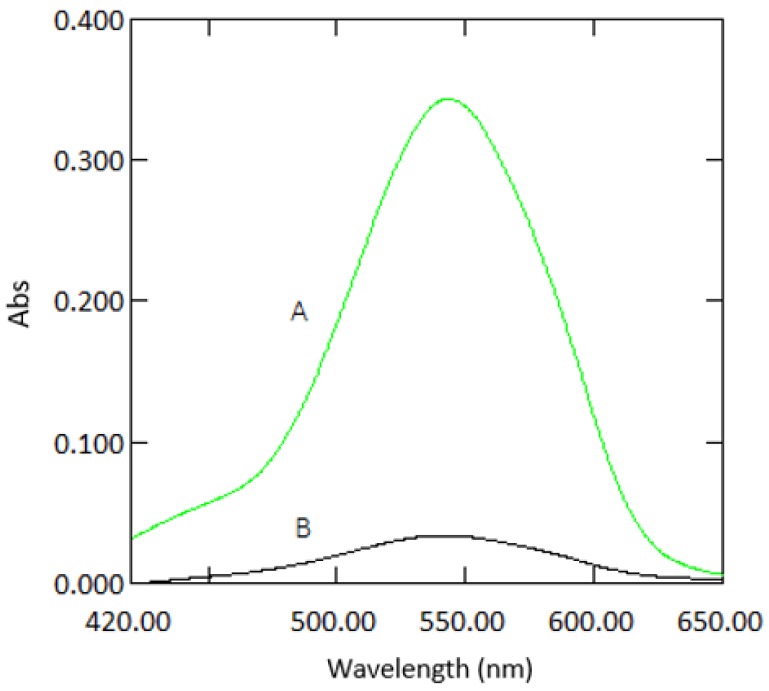
Absorption spectra of a sample containing 1 mg·L^−1^ Cr VI with reagents measured in 10 mm cuvettes (**A**) and 1 mm quartz cuvettes (**B**) against reagent blank.

**Figure 3:**
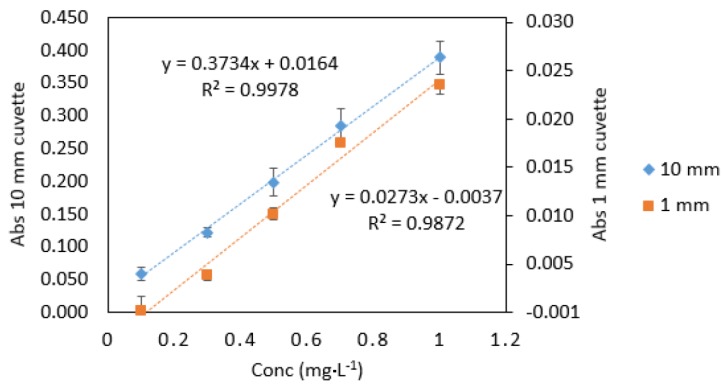
Comparison of Cr VI standards (0.1–1 mg·L^−1^) measured in quartz cuvettes with 10 mm and 1 mm path lengths. All measurements were carried out in triplicate (*n* = 3).

As expected, the absorbance values and the slope for 1 mm quartz cuvette measurements were 10 times lower than those obtained from 10 mm standard cuvette measurements (Figure 2). The analytical response was strong for samples measured in microcuvettes, as can be seen from the calibration graphs (Figure 3). The good response signal and the linearity obtained from microcuvette measurements strongly indicate that the DPC method is applicable for use in microfluidic detection systems.

### 3.2. Sample Cell Cleaning Validation

Sample cell cleaning is important for residue removal from previous analysis that can otherwise cause low sensitivity and lack of precision. Ideally the cleaning method should be time-efficient and simple. The most effective solvent for quartz cuvette rinsing was proven to be 1% nitric acid as it removed all the stains caused by the DPC method’s colour reaction, whereas methanol was found to be the least effective (Table 2). The 1% nitric acid could be applied for rinsing sample cells in microfluidic detection systems. 

### 3.3. Optimisation of Parameters

#### 3.3.1. pH

**Figure 4:**
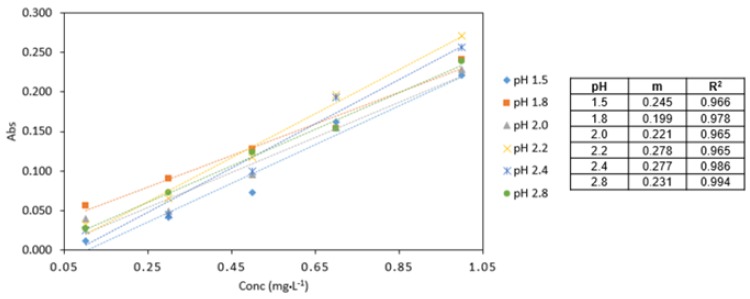
Comparison of Cr VI (0.1–1 mg·L^−1^) analysed at various pH conditions (pH 1.5, 1.8, 2.0, 2.2, 2.4, 2.8). All measurements were carried out in triplicate (*n* = 3).

The highest absorbance values were obtained at pH 2.2 (Figure 4). The analytical response pH 2.2 was found to be the optimum pH for the procedure and used in subsequent experiments. 

#### 3.3.2. Sample/Reagent Ratio

**Figure 5:**
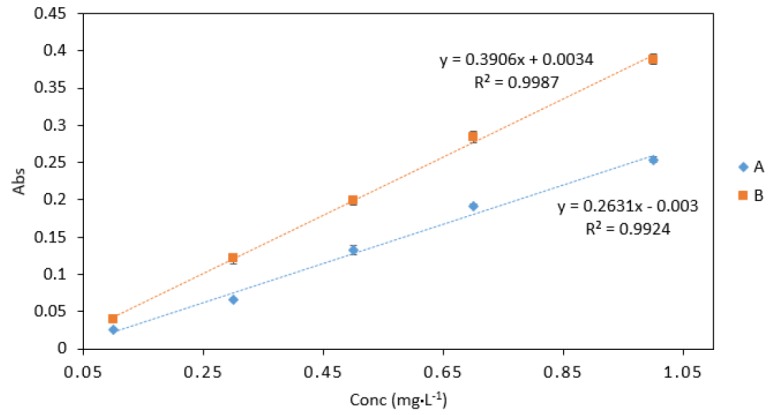
A comparison of Cr VI (0.1–1 mg·L^−1^) analysed using two sample/reagent ratios: (**A**), 2 (Cr): 2 (0.4 M sulphuric acid): 2 (DPC) and (**B**), 2 (Cr): 2 (0.4 M sulphuric acid and DPC mix). All measurements were carried out in triplicate (*n* = 3).

Sample/reagent ratio B gave the best response with higher absorbance values than sample/reagent ratio A (Figure 5). The slope obtained from ratio B was also higher than that of ratio A. Therefore, ratio B was chosen for use in microfluidic detection systems. Furthermore, ratio B requires a small number of separate reagents, which allows for cost efficient fabrication and a simple microfluidic design.

#### 3.3.3. Reagent Stability

**Figure 6:**
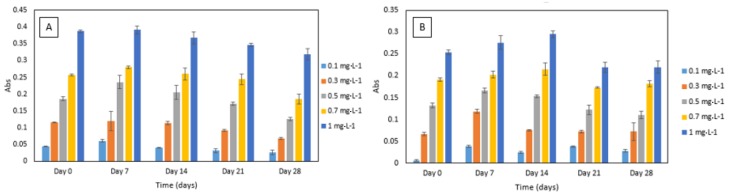
(**A**) Stability of 1,5-diphenylcarbazide (DPC) dye in Cr VI (0.1–1 mg·L^−1^) analysed periodically over day 0, 7, 14, 21 and 28. All measurements were carried out in triplicate (*n* = 3); (**B**) stability of sulphuric acid and DPC dye mixture in Cr VI (0.1–1 mg·L^−1^) analysed periodically over day 1, 7, 14, 21 and 28. All measurements were carried out in triplicate (*n* = 3).

For the DPC dye stability experiment, an increase in absorbance was noted after seven days. After that, decreasing absorbance values over time were noted (Figure 6). A similar trend was observed for DPC dye and sulphuric acid reagent mixture’s stability experiment. The absorbance increased after seven to 14 days and then decreased over time (Figure 6). Furthermore, the method yielded analytically useful calibration data over the time period studied and showed good potential for application in a microfluidic analysis system. Regular calibration protocol should be implemented for correcting the change in absolute absorbance values.

#### 3.3.4. Effect of Different Acid Concentrations

**Figure 7:**
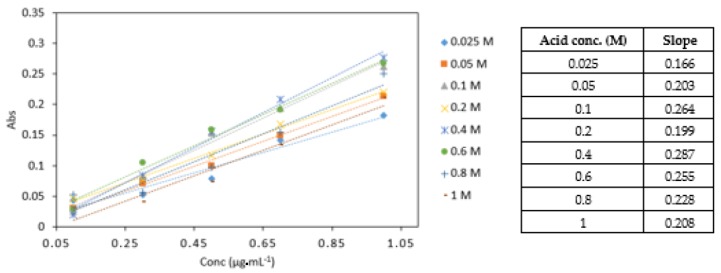
A comparison of Cr VI (0.1–1 mg·L^−1^) analysed with various sulphuric acid concentrations (0.025, 0.05, 0.1, 0.2, 0.4, 0.6, 0.8 and 1 M). All measurements were carried out in triplicate (*n* = 3).

0.4 M sulphuric acid yielded the highest absorbance values and the highest slope in comparison to other acid concentrations analysed (Figure 7). The statistical analysis showed that there was a significant difference between the different acid concentrations (*p* < 0.05). No significant difference was found between 0.4, 0.6, and 0.8 M acid concentrations. The 0.4 M sulphuric acid was used in subsequent experiments. 

### 3.4. Colour Stability

**Figure 8:**
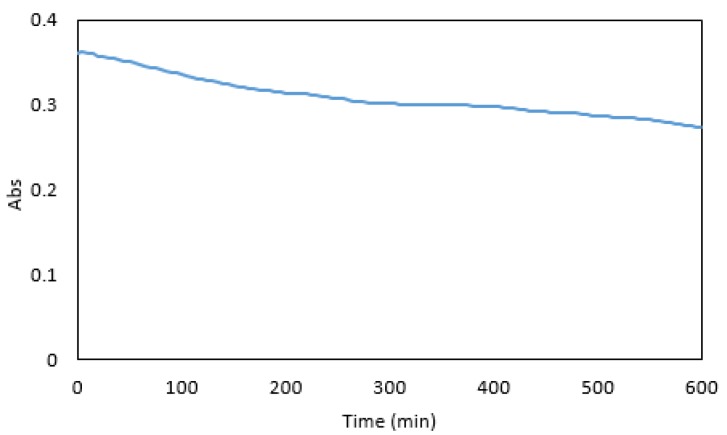
Absorbance of 1 mg·L^−1^ Cr VI premixed with the reagents over 600 min.

Maximum absorbance was reached five minutes after the addition of the dye (Figure 8). At this time the absorbance was sufficiently stable for measurements, and a five-minute reaction time was used in subsequent experiments. After 600 min, a 24% decrease in absorbance was observed. Overall, the colour stability was good and suitable for measurements in a microfluidic detection system.

### 3.5. Interference

From the different species investigated, Fe (III) interfered most strongly with the DPC method (Table 3). Iron interference was masked by 1% ascorbic acid. Slight interference was observed from Cr (III), Mn, Mg, and NO_3_. In general, Cr (III) and Mn concentrations in surface water would be expected to occur below the tolerance limit and would not pose any interference with the optimised DPC method [58]. However, the method’s effectiveness would be affected in surface waters with high magnesium and nitrate levels and should be taken into account when designing calibration protocols for detection devices [59]. 

### 3.6. Environmental Water Samples

**Figure 9:**
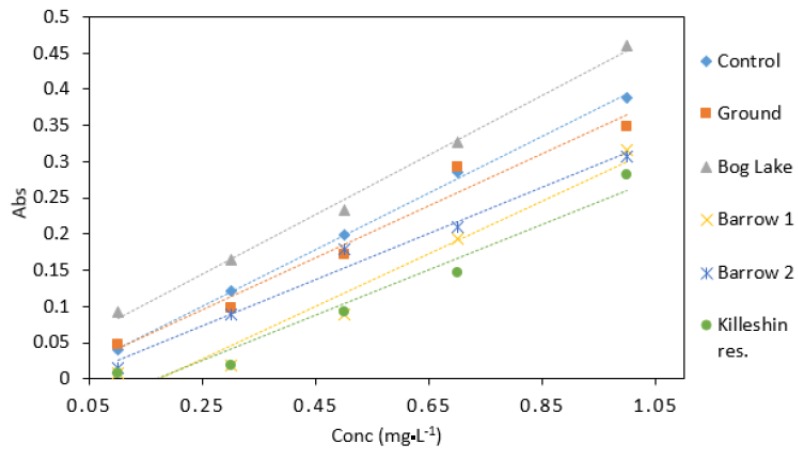
Comparison of Cr VI (0.1–1 mg·L^−1^) analysed in several water matrices. All measurements were carried out in triplicate (*n* = 3).

Groundwater samples had a similar response to the control samples (Figure 9). This would indicate that groundwater samples did not contain high amount of interfering substances. The highest absorbance values were observed in samples collected from Bog Lake. The lowest absorbance was obtained from Killeshin reservoir. The difference in absorbance values could be explained by factors such as sample colour and chemical composition. Overall, different water matrices have the potential to affect the result of the DPC method. Despite the robust results shown in Table 4, this should be considered for calibration protocol development in microfluidic detection devices. 

### 3.7. Comparison between Optimised DPC Method and ICP-MS 

Evaluation of Cr VI in different water samples using ICP-MS and the optimised colorimetric DPC method showed that the Cr VI concentrations are comparable (Table 4). The largest percentage difference between ICP-MS and the optimised method was observed for the control sample as 14.6%, whereas the smallest percentage difference was found for the groundwater sample as difference of 7.5% was obtained. Therefore, it can be concluded that the optimised DPC method is effective in terms of Cr VI determination in various water matrices.

### 3.8. Analytical Data

**Figure 10:**
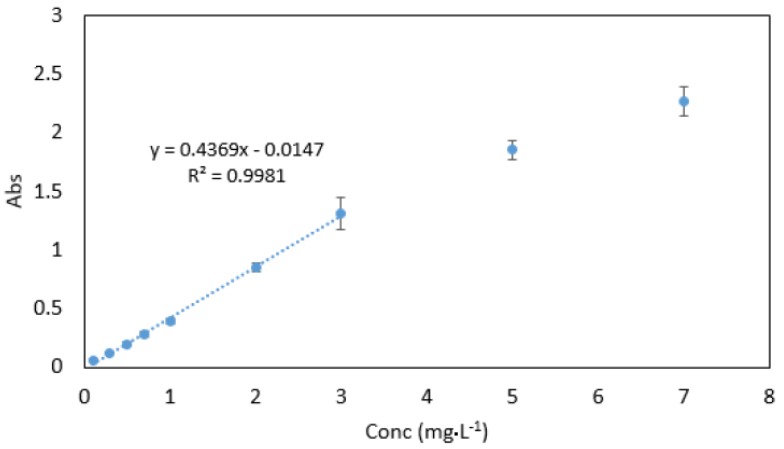
Calibration curve for Cr VI ranging between 0.03–7 mg·L^−1^. All measurements were carried out in triplicate (*n* = 3).

**Figure 11:**
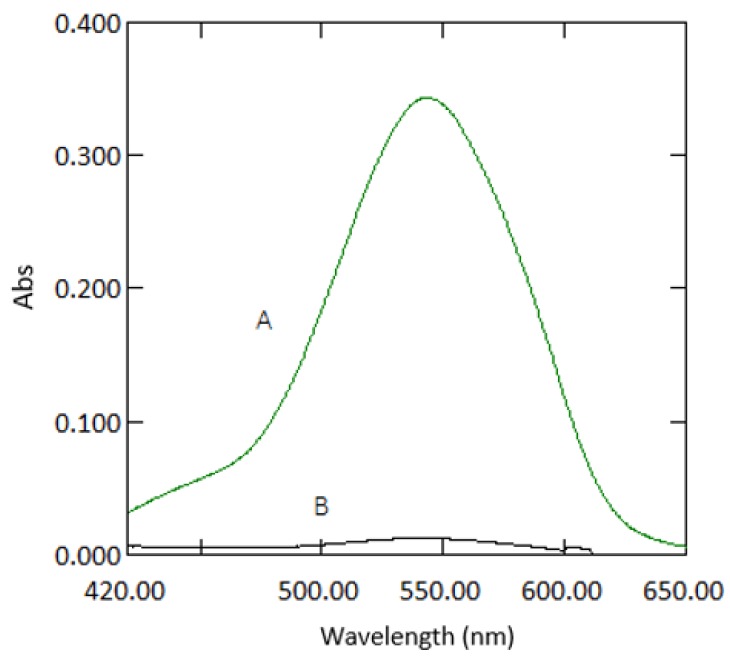
Absorption spectra of coloured species (1 mg·L^−1^ chromium) versus reagent blank (**A**) and reagent blank versus double deionised water (**B**).

Beer’s law was obeyed in the range between 0.03–3 mg·L^−1^ (Figure 10). The molar absorptivity coefficient was found to be 2.021 × 10^4^ mol^−1^·cm^−1^. Sandell’s sensitivity was found to be 2.574 × 10^−3^ µg·cm^−2^. The limit of detection (3 sdb·m^−1^) and the limit of quantification (10 se·m^−1^) (where *sdb* is the standard deviation of the reagent blank, and *m* is the slope of the calibration curve) were found to be 0.023 and 0.076 mg·L^−1^, respectively. Absorption spectra of 1 mg·L^−1^ Cr VI against reagent blank and reagent blank against double deionised water are shown in Figure 11.

## 4. Discussion

The results showed the optimised DPC method was more sensitive than some of the previous studies [53,55,61] (Table 5). Although the Cr VI determination method proposed by Amin et al. yielded very low detection limit [62], the ADTP reagent had to be synthesised in the laboratory prior to the analysis. One of the advantages associated with the optimised DPC method is that the reagents are commercially available. Wang et al. used gold nanoparticles to develop a method for Cr III and Cr VI determination in water with a detection limit of 0.001 mg·L^−1^ for Cr VI [63]. However, there are several drawbacks associated with gold nanoparticle synthesis and cost [64]. The simplicity of the method and the relatively wide linear range indicates that the proposed method is suitable for use in autonomous microfluidic detection systems and Cr VI determination in chromium-containing effluents and environmental waters.

## 5. Conclusions

The DPC method showed great potential for use in autonomous microfluidic detection systems for Cr VI detection in water. The method was optimised for incorporation into micro scale detection systems. The method proved to be simple, fast, and robust. Strong analytical response was obtained from 1 mm light path cuvettes, demonstrating that the method would be effective in a small-scale detection system. Furthermore, the optimised method required a small number of reagents, resulting in cost effective analysis. Strong analytical response was obtained from a simple 1:1 sample/reagent ratio. The reagent mixtures were stable for two weeks, with a gradual decrease in absorbance observed after that. Investigation of the method’s performance in different water samples and the good agreement obtained with ICP-MS measurements revealed that the method is suitable for determination of Cr VI in various water matrices. The optimised method has potential for Cr VI monitoring applications in surface and waste water using microfluidic detection systems.

## Figures and Tables

**Table 1:** Ion-coupled plasma mass spectrometer (ICP-MS) settings.

Instrumental Parameters	Scanning Parameters
Plasma flow: 15 L·min^−1^	Scanning mode: Peak hopping
Auxiliary flow: 1.55 L·min^−1^	Number of replicates: 3
Nebuliser flow: 0.9 L·min^−1^	Pump rate: 9 rpm
Sheath gas flow: 0.2 L·min^−1^	Rinse time: 40 s
Sampling depth: 6.5·mm	Sample uptake delay: 50 s
Power: 1.4 kW	Internal standards: Li^6^, Sc^45^, Y^89^, Tb^159^, Ho^165^, Th^232^

**Table 2:** A comparison between absorbance values of quartz cuvettes rinsed with different solvents.

Solvent	Abs	Abs	Abs	Average	SD	% RSD
Water	0.012	0.018	0.014	0.015	0.003	20.830
1% HCl	0.041	0.017	0.081	0.046	0.032	69.780
1% HNO_3_	0.004	0.003	0.001	0.003	0.002	57.282
Methanol	0.088	0.101	0.105	0.098	0.009	9.070
Acetonitrile	0.021	0.022	0.033	0.025	0.007	26.283
Acetone	0.085	0.081	0.086	0.084	0.003	3.150
Tween 20	0.013	0.012	0.007	0.011	0.003	30.136
1% H_2_SO_4_	0.006	0.005	0.006	0.006	0.001	10.189
Control	0.002	0.001	0.002	0.002	0.001	34.641

**Table 3:** Effect of foreign species on the determination of Cr (VI) (1 mg·L^−1^).

Interferents	Tolerance Limit (mg·L^−1^)
Fe (III)	1
Cr (III), Mn, Mg, NO_3_	10
PO_4_	100

**Table 4:** A comparison between Cr VI concentrations calculated using the optimised DPC method and measurements obtained from an accredited ICP-MS.

Sample	ICP-MS Unspiked Sample (mg·L^−1^)	ICP-MS (mg·L^−1^)	DPC Method (mg·L^−1^)	Percentage Difference (%)
Control	0.000	0.883	1.012	14.607
Ground	0.002	0.930	1.000	7.544
Killeshin res.	0.002	0.959	1.063	10.805
St Mullins	0.001	0.987	1.101	11.595
Bog Lake	0.001	1.060	0.960	9.467
Barrow	0.002	0.907	1.024	13.249

**Table 5:** Comparison of spectrophotometric methods of the Cr VI determination.

Detection Principle	ʎ_max_ (nm)	LOD (mg·L^−1^)	Linear Range (mg·L^−1^)	Reference
µPDAs	453	0.041	0.041–0.072	54
µPDAs	530	30.000	40.000–400.000	53
Rotational µPDAs	445	0.180	0.500–10.000	55
Gold nanoparticles	520	0.001	0.010–0.130	63
Spectrophotometric	503	0.030	0.010–0.400	62
Spectrophotometric	385	0.014	0.260–26.000	59
Spectrophotometric	543	0.023	0.030–3.000	This study
